# The impact of the COVID-19 pandemic on the lifestyle and behaviours, mental health and education of students studying healthcare-related courses at a British university

**DOI:** 10.1186/s12909-022-03179-z

**Published:** 2022-02-21

**Authors:** Nishita Gadi, Saman Saleh, Jo-Anne Johnson, Aaron Trinidade

**Affiliations:** 1 Anglia Ruskin University Medical School, Chelmsford, UK; 2 Southend University Hospital NHS Foundation Trust, Department of Otolaryngology, Southend University Hospital, Prittlewell Chase, Southend-on-Sea, SS0 0RY UK

**Keywords:** COVID-19, Medical student, Medical education, Anxiety, Pandemic

## Abstract

**Background:**

The COVID-19 pandemic has affected most industries, including health education. In this study, we surveyed students studying healthcare-related courses at our university on how their lifestyles and behaviours, mental health and education had been affected by the pandemic.

**Methods:**

Mixed methods cross-sectional study.

**Results:**

Two hundred thirty-three students responded to the questionnaire. Lifestyle and behaviours: 51.5% of the participants changed their diet (*n*=120); 45.5% (*n*=106) exercised less; 66.5% (*n*=155) experienced a change in sleep; 51.1% (*n*=119) reported a change in appetite. Mental health: 84.2% (*n*=196) reported worrying too much about different things; 61.9% (*n*=144) could not stop or control worrying; 71.2% experienced trouble relaxing on several days or more (*n*=166). At least sometimes, 72.1% (*n*=168) felt unable to cope with things they had to do; 8.5% (*n*=20) never, or almost never, felt confident about handling personal problems. Education: 65.7% (*n*=153) struggled to complete learning outcomes with online delivery; 82% (*n*=191) worried about practical skills being affected; 60.5% (*n*=141) worried about the impact of COVID-19 on their future career. Almost half (48.9%, *n*=114) believed that online teaching should be part of the standard curriculum.

**Conclusion:**

In general, there was a negative impact on behaviours, lifestyle and mental health and virtual education was perceived as necessary in making up for the loss of face to face experiences. Students’ mental health and educational needs have been affected by the current pandemic and healthcare educational facilities must respond to these needs to ensure students continue to receive the support they need.

**Supplementary Information:**

The online version contains supplementary material available at 10.1186/s12909-022-03179-z.

## Introduction

The SARS-Cov-2 virus (COVID-19) outbreak began in early December 2019 [1]. On the 11^th^ of March 2020, the World Health organisation (WHO), classified COVID-19 as a pandemic and by November 2020, there were 53,164,803 confirmed cases and 1,300,576 deaths globally [2, 3]. In the UK, this has had an unprecedented social impact with the government enforcing rules such as lockdowns in an attempt to control its spread [4]. Education and healthcare industries have been particularly affected, and there has been a negative impact on mental health. A British study by Brooks, *et al.* (2020) reported negative psychological effects, such as post-traumatic stress disorder symptoms, confusion and anger, when reviewing the psychological impact of quarantine [5].

We had noticed an anecdotal rise in the levels of such negative emotions and also lifestyle and behavioural changes amongst students in our medical school, findings reflected in other medical schools around the UK, with the sentiment that the pandemic was no time to be a student [6]. On questioning course leaders from other parts of the University’s Healthcare Faculty (e.g. nursing and paramedical courses), the sentiment was broadly the same. This was in keeping with several international reports of the negative impact that the pandemic was having on medical students in other, but not all, parts of the world [7–14].

Frontline health-care workers are a demographic particularly susceptible to the psychological consequences of the pandemic [11] and healthcare students share many of the same risk factors. With the closure of universities, healthcare students, as with other students, have been experiencing problems such as interruptions to their education, loss of peer support networks, and uncertainty in volunteering in hospitals during the crisis [12–14].

The primary objective of this study was to investigate whether our anecdotal findings of the COVID-19 pandemic negatively affecting healthcare students at our university was true and whether these findings were in line with those found at other centres around the world. To do this, we surveyed undergraduate and postgraduate students studying healthcare-related courses on how their lifestyles and behaviours, mental health and education has been affected by the pandemic. Findings of this study will be used to inform appropriate student support strategies.

## Methodology

### Overview

In this cross-sectional study, participants were asked to complete an online survey. Students enrolled in courses categorised under the Faculty of Health, Education, Medicine & Social Care (HEMS), at the host university were included in the study. At the time of the questionnaire, a total of 3633 students were enrolled in the HEMS, including the Schools of Medicine (*n*=325), Nursing & Midwifery (*n*=106), Allied Health (*n*=2000), and Education & Social Care (*n*=630).

There were no exclusion criteria.

The survey was open and distributed to students between 4^th^ September 2020 and 5^th^ November 2020. During this period, the first UK National lockdown restrictions were eased. Social distancing rules, such as the ‘rule of six’ were initiated. In October, a three-tiered system was started in England. Universities reopened but most of the teaching remained online. At our university, healthcare students had a mixed online and face-to-face teaching schedule, with the majority being online. The university was prepared for online study.

The online surveys consisted of four sections: (1) demographics; (2) lifestyle and behaviours; (3) mental health; (4) education. The survey asked students to answer these questions based on how the pandemic had influenced them at the time of the survey and during previous lockdowns.

### Demographics

The demographic data included course of study, year of study, gender, age, ethnicity and religion. As gender and religion are special category data, the "Ethnic group, national identity, and religion" guide by The Office of National Identity was used to formulate the answer options for these questions [15].

### Lifestyle and behaviours

The lifestyle and behaviours section contained questions based on a similar survey by Gallè, *et al*. (2020), which aimed to understand the knowledge and behaviours related to the COVID-19 epidemic in Italian undergraduate students [9].

### Mental health

The Mental Health section of the questionnaire were structured to provide descriptive data with an evaluative character and considered symptoms of mental health disorders. The Depression Anxiety Stress Scales-21 (DASS-21) was used as a guide to formulate the questions.

### Education

The education section was constructed by considering changes in education delivery during the pandemic; questions were asked to investigate the impact of these changes. At the time of questionnaire delivery, most face to face lectures had been replaced by on-line lectures and resources.

### Open comments

At the end of each section, students were invited to provide free-text open comments.

### Statistical Analyses

Data was analysed using SPSSv27 and Excel version 16.16.9. In this mixed methods study, quantitative data analysis was used to interpret closed question survey responses in each of the three categories. Qualitative analysis was used to interpret the free-text responses in each of the three categories. In order to analyse the qualitative data a thematic analysis was done: after reading through all the responses, common themes were found within the three categories (lifestyle and behaviours, mental health and education); all the responses were coded accordingly, and the frequency of each theme was obtained.

## Results

A total of 233 out of 3633 students completed the survey resulting in a 6.4% response rate. With respect to medical students specifically, the largest cohort, the response rate was 16% (*n*=52/325). Postgraduate students comprised 8.2% (*n*=19/233) of the cohort.

**Fig. 1:**
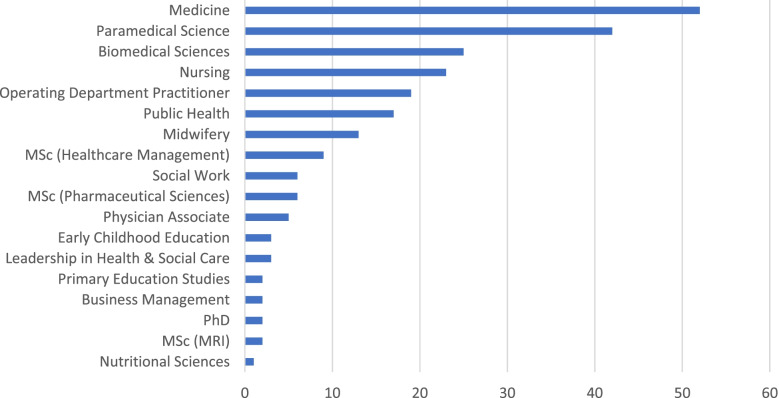
Bar-chart showing the breakdown of respondents by course

Of the total 233, respondents were predominantly female (F:M = 3:1; F=179, M=54), identified as white British (*n*=127, 54.4%) and had no religious belief (*n*=95, 40.8%). The mean age was 26.8 ± 9.69 (Table 1). The majority of respondents were from the school of medicine (*n*=52/233, 22.4%), followed by paramedical science (*n*=42/233, 18.1%) and biomedical sciences (*n*=25/233, 10.8%). In all, 18 courses were represented (Fig. 1).

**Table 1:** Demographic data of questionnaire respondents

Category	Demographic	
		**Mean ± SD**
**Age (Years)**		26.8 ± 9.69
		**n (%)**
**Gender**	Male Female	54 (23.2%) 179 (76.8%)
**Religion**	Muslim No religion Hindu Christian Sikh Buddhist Other	21 (9%) 95 (40.8%) 16 (6.9%) 93 (39.9%) 3 (1.3%) 1 (0.4%) 4 (1.7%)
**Ethnicity**	Arab White: British Asian: Indian Asian: Pakistani Asian: Chinese Black: African Other Asian: Bangladeshi White: Irish Black: Caribbean	7 (3%) 127 (54.5%) 21 (9%) 6 (2.6%) 5 (2.1%) 31 (13.3%) 32 (13.7%) 0 (0%) 2 (0.9%) 2 (0.9%)

### Quantitative analysis

#### Lifestyle & behaviours

More than half of the participants saw a change in their diet (*n*=120; 51.5%), with more people experiencing a worsening of their diet (*n*=72; 30.9%) rather than an improvement. Additionally, 45.5% (*n*=106) of participants exercised less. In terms of smoking, most students (*n*=201; 86.3%) did not smoke prior to the lockdown; 5.6% (*n*=12) of the participants did alter their smoking behaviour, with an equal number either stopping smoking, or starting to smoke during the pandemic.

**Fig. 2:**
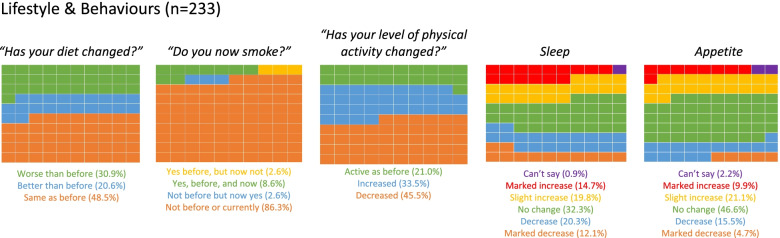
Waffle chart showing student responses to the Lifestyle & Behaviours questionnaire

Approximately two-thirds of the participants (*n*=155; 66.5%) experienced a change in sleep and 51.1% (*n*=119) reported a change in a change in their appetite. Changes in both sleep and appetite were most commonly seen to decrease rather than an increase (Fig. 2).

#### Mental Health

The levels of the five emotions investigated (sadness, irritability, fatigue, frustration and loneliness) were shown to either slightly increase or not change in most people. An increase in each emotion was more common than a decrease.

The following feelings were experienced by more than half of the students: anxiety, stress, worry, irritability, fear, and difficulty relaxing. Most commonly, 84.2% (*n*=196) of students reported worrying too much about different things, with 21.9% (*n*=51) experiencing this nearly every day, and 61.9% (*n*=144) not being able to stop or control worrying. Most participants experienced trouble relaxing on several days or more (*n*=166; 71.2%).

**Fig. 3:**
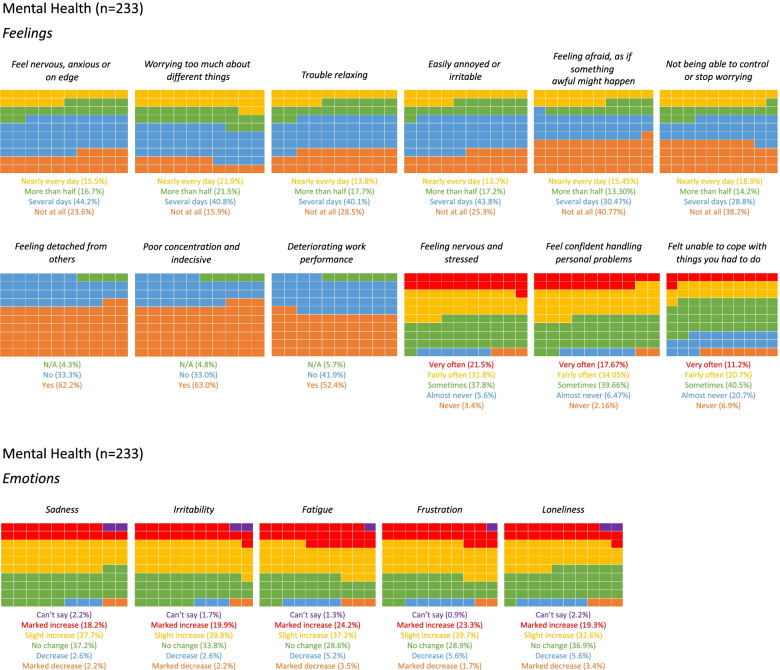
Waffle chart showing student responses to the Mental Health questionnaire, subdivided into (**a**) feelings and (**b**) emotions

Moreover, most students reported feeling detached from others (*n*=145; 62.2%), having poor concentration and being indecisive (*n*=143; 61.4%), and experienced deteriorating work performance (*n*=120; 51.1%). At least sometimes, 72.1% (*n*=168) felt unable to cope with things they had to do; 8.5% (*n*=20) never, or almost never, felt confident about handling personal problems (Fig. 3a & b).

#### Education

About 44.2% (*n*=103) did not agree that they were provided with all the information needed to continue academic studies, and 60.1% (*n*=140) did not agree that their academic studies have been adequately supported since the start of the pandemic.

The majority of students agreed with the following statements: they struggle to complete learning outcomes with online delivery (*n*=153; 65.7%), they are worried that their practical skills will be affected (*n*=191; 82%) and they are worried about the impact of COVID-19 on their future career (*n*=141; 60.5%). Eighteen percent of students (*n*=42) had even considered leaving their course.

**Fig. 4:**
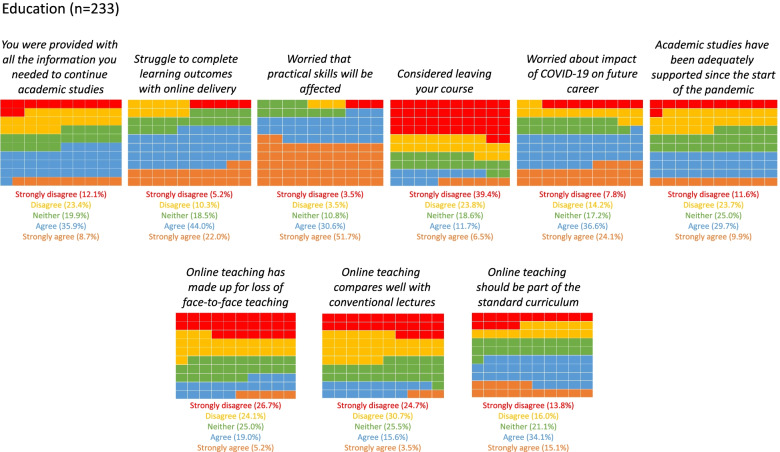
Waffle chart showing student responses to the Education questionnaire

In terms of views regarding online teaching, more students disagreed (*n*=118; 50%), rather than agreed (*n*=36; 24.1%), that online teaching had made up for face-to-face teaching, such as conventional lectures or practical skills teaching. Many participants (*n*=187; 80.3%) did not agree that online teaching compared well to conventional lectures. However, 48.9% (*n*=114) believed that online teaching should be part of the standard curriculum (Fig. 4).

#### Qualitative analysis

A total of 66 participants provided at least one free-text open comment at the end of each section (excluding the demographics section). There were 26 lifestyle and habits comments, 22 mental health comments and 33 education comments. A coding system was used to search for trends in comments (Table 2). The comments given reflected the findings of the quantitative analysis. Specific findings included the role the pandemic played in aggravating underlying mental health issues in some students and the frustration voiced over the perceived disruption in course delivery which has been only partly addressed by virtual learning.

**Table 2:** Qualitative analysis of open-answer questions

Theme	Codes	n	Extracts
**Lifestyle & habits**	Lifestyle and habits worsened because of pandemic.	13	*“Very tired, no energy, been at home for so long, now finding it extremely overwhelming coming back to placement.”* *“Due to not wanting to share the train carriages with others I have begun cycling (15 miles) to get to my part time job.”* *“Levels of inactivity/activity fluctuated quite dramatically throughout the pandemic”* *“Consuming more alcohol than before and eating fast foods.”*
	Lifestyle and habits improved because of pandemic. Lifestyle and habits fluctuated	10 2	
	Lifestyle and habits affected by non-pandemic factors.	1	
**Mental Health**	Mental health issues caused by pandemic	10	*“I have a history of depression and anxiety but had been coping well prior to lockdown. However, due to the pressures of lockdown, I had to be prescribed anxiety medication once again, which I continue to take.”* *“I would say it is the first time that I have felt significantly anxious. My sleep was heavily affected at the beginning of the pandemic. I think it was due to their being so many unknowns e.g. how bad this will be, how will my children be affected, when will I be able to see my family. My studies I would say were more towards the bottom of my 'worry list' which I think [reflects] the good support we got from ARU. I felt the university did the best they could in such unprecedented times.”* *“ … lock-down and the stress of my PhD caused me* *to develop moderate anxiety & depression. I have since sought support from the well-being service.”*
	Pre-existing mental health condition worsened by pandemic.	5	
	Mental health improved during pandemic.	4	
	Mental health worsened by factors other than pandemic.	3	
**Education**	Educational disruption; lack of guidance.	15	*“Online teaching resources have been adequate but can't in any way make up for the practical placement I missed out on. I also feel there has been a lack of guidance on returning to placement and what may happen in the future with placement for students if there is a second wave.”* *“We received no support, very little teaching or communication and were left to try to learn what we needed to know with no guidance. As a result, many of the class failed elements of the course and have to retest causing stress and anxiety as well as preventing us from progressing in our careers.”*
	Worry about missing practical and interactive sessions.	7	
	Satisfaction with virtual teaching.	7	
	Education not affected by pandemic.	4	

Nearly all of the comments in the lifestyle and behaviours demonstrated that the pandemic influenced their behaviour (*n*=23/26, 88%), with more people stating the pandemic influenced a negative behavioural change (*n*=13/26, 50%) versus a positive behaviour change (*n*=10/26, 38%). Similarly, the majority of answers in the Mental health section state the pandemic caused or exacerbated mental health issues (*n*=15/22, 68%).

With respect to the education section open comments, the most common concerns were related to educational disruption and lack of guidance (*n*=15/33, 45%). There was also worries about missing practical sessions (*n*=7/33, 21%). Conversely, there were a few answers which show satisfaction with virtual teaching (*n*=7/33, 21%), and some that state their education was not affected by the pandemic (*n*=4/33, 12%).

## Discussion

### The primary message

Our study indicates that the COVID-19 pandemic has had an impact on the lifestyle, behaviours, emotions, feelings, and educational experiences of healthcare students at our university. Results show both negative and positive impact, however, negative change was more commonly seen, including decreased physical activity, worsened diet, increased negative emotions and feelings, and perceived disruptions to education with the shift to online delivery.

### The perspective

It is well-documented that the prevalence of depression, depressive symptoms and suicidal ideation is higher amongst medical students, as reported in the pre-COVID-19 era in a systematic review of the literature and meta-analysis by Rotenstein, *et al.* (2016) [16]. However, since the pandemic, self-reporting by medical students, as in our study, have suggested a further increase in these conditions. The mental health findings in our study are consistent with other recent international studies. In an Indian longitudinal prospective study of 217 medical students, Saraswathi, *et al.* (2020) found that the COVID-19 pandemic appeared to negatively affect the mental health of their students with the prevalence and levels of anxiety and stress being increased but depression symptoms remaining unaltered [10]. In a Chinese-based large cross-sectional, survey-based, region-stratified study by Ye, *et al.* (2020), demographic data and mental measurement was collected from 2,498 medical students and 1,177 non-medical students in 31 provinces. They found that medical students suffered from more stress than non-medical students almost in all provinces of China [11]. Conversely, in a cross-sectional study of Iranian medical students by Nakhostin-Ansari, *et al.* (2020) found that whilst depression and anxiety were prevalent during the pandemic, they did not significantly differ among their students before and after the COVID-19 outbreak. They surmised that minimizing students' presence in the clinical settings and their exposure to COVID-19 patients may have helped control anxiety symptoms [12].

Studies on nursing students have revealed similar findings. In a cross-sectional study of 586 nurses in eastern China study by Wang, *et al.* (2021) The prevalence of nurses' anxiety and depression during the pandemic was 27.6% and 32.8%, respectively [13]. A large survey study of 1485 medical and dental students in the United Arab Emirates by Saddik, *et al.* (2020) showed that medical students have lower levels of anxiety during the pandemic than dental students, although this became higher during clinical rotations and decreased once virtual learning was introduced [14]. But despite these pandemic-related anxieties, studies show a high level of interest in and understanding about COVID-19 and healthcare students are still seen as important ambassadors and activists for infection control measures, especially in countries where medical education has been suspended, with many recognising their responsibility to their communities as volunteers and the importance and risks of their chosen professions [17, 18].

There are few studies that specifically examine changes in lifestyle and habits as a result of the pandemic, but none in healthcare students. In a Saudi Arabian cross-sectional on-line survey of 280 children aged 6-15, Hanbazaza & Wazzan (2021) provide evidence for the negative influences of the COVID-19 curfew on health behaviours, including eating habits, physical activity, and sedentary behaviour [19]. Similarly, in an on-line questionnaire of 1048 South African adults, Lewis, *et al.* (2021) showed in their population that COVID-19-related low physical activity predicted greater insomnia symptom severity, which in turn predicted increased depressive and anxiety-related symptoms. Overall, relationships between the study variables and mental health outcomes were amplified during lockdown. The findings highlight the importance of maintaining physical activity and reducing sedentary screen-use to promote better sleep and mental health [20]. Our study reflects these changes with most students reporting a worsening of their diet and a reduction in the amount of physical exercise they took. It is likely, therefore, that these changes were linked to their increased anxiety and depression levels.

In terms of education, closure of schools and universities, including all UK medical schools, has led to the cessation of live lecturers in a conventional lecture theatre and the cancellation of clinical placements. However, there have been innovative methods of delivering education to minimise this disruption to training, with a shift towards teleteaching including online webinars on various platforms such as Zoom (Zoom Video Communications, Inc., San Jose Ca, USA) and online open-book examinations [21]. Virtual and e-learning-based medical education, including multimedia study materials and virtual surgical electives, has proven extremely vital to provide an acceptable education for the undergraduate medical students during an outbreak to ensure that the maintenance of satisfactory education standards [22–24]. Service learning, a form of experiential education that is being implemented internationally within undergraduate primary care, with the potential to significantly enhance clinical practice whilst simultaneously facilitating medical students' learning, is also an example of a new learning model that will continue to gain traction in the post-COVID era [25].

Generally, our results showed that medical students did not feel that their education was as negatively impacted as other students. Interestingly, a large proportion of medical students strongly disagreed with ever considering leaving their course whereas the responses from other students was more equally distributed across all options. This difference might be explained by differences in motivational and learning strategies in medical students whose focus tends to be on lifelong learning [26, 27]. The majority of medical students also seemed to disagree that online teaching had made up for face-to-face teaching but agreed that there was still a role for online teaching within the standard curriculum. The general consensus for other students was less clear.

It was clear from our data that students at our institution are struggling on different levels. Whereas previously, academic studies could be supplemented with collaborative learning or a relaxing social life at university, the COVID-19 pandemic along with social distancing regulations, has resulted in a very isolated lifestyle. Healthcare students are particularly susceptible to these changes as their education depends on interpersonal interactions, in the form of placements and clinical skills sessions. The restrictive measures imposed on healthcare students owing to the threat of COVID-19 have robbed many of the joys of university life [6]. More must be done to ensure these students are adequately supported during the lockdown, such as ensuring the availability of robust and imaginative learning opportunities for students, providing opportunities to help with the pandemic response and ensuring access to pastoral care. Dyrbye, *et al.* (2015) show that amongst medical students, only a third with burnout will seek help due to the perceived associated stigma, negative personal experiences, and the hidden curriculum all contributing [28]. From our data, we suggest that may be true for students in all healthcare disciplines at our facility.

### Limitations of this study

This study is limited to data from a single university and therefore only reflects the population of students present at this particular institution. However, recently published studies investigating the same variables at different institutions and countries, show similar findings to this paper, and anecdotally, these sentiments have been expressed by students in other universities both in the press and on social media platforms.

We studied a heterogenous sample with varying participant ages and educational backgrounds, which we believe makes our results more generalisable. The participant characteristics may have influenced the impact the pandemic had on them, for example, being a postgraduate student may have allowed them to deal with stress better. Separating the data or performing sub-group analyses would have allowed us to identify these variables but given the relatively small numbers, we decided to keep our analysis to the group as a whole.

The response rate was low, but comparable with other published studies and we feel that it still provides an extremely useful snapshot of the effect the pandemic had on the student body.

This study did not ask the students to justify their emotions or feelings, nor did it investigate what the students were doing during the pandemic, such as if they were volunteering in a healthcare setting, or if they were in lockdown by themselves or with others. These factors may have impacted their survey responses.

## Conclusion

Our study suggests that the COVID-19 pandemic has generally had a negative impact on the mental health, perceived education and lifestyle of healthcare students. It also highlights areas where universities delivering healthcare education can focus resources to improve their students’ wellbeing and educational needs. Such improvements are likely to have long-lasting benefits in the post-COVID era.

## Supplementary Information

**Additional file 1.**

**Additional file 2.**

## Data Availability

All data generated or analysed during this study are included in this published article and its supplementary information files.

## References

[1] Harapan H, Itoh N, Yufika A, Winardi W, Keam S, Te H (2020). Coronavirus disease 2019 (COVID-19): A literature review. J Infect Public Health..

[2] https://www.who.int/publications/m/item/weekly-operational-update-on-covid-19%2D%2D-13-november-2020. Last accessed 15 Feb 2021

[3] https://www.who.int/emergencies/diseases/novel-coronavirus-2019/events-as-they-happen. Last accessed 15 Feb 2021

[4] Iacobucci G (2020). Covid-19: UK lockdown is "crucial" to saving lives, say doctors and scientists. BMJ..

[5] Brooks SK, Webster RK, Smith LE, Woodland L, Wessely S, Greenberg N, Rubin GJ (2020). The psychological impact of quarantine and how to reduce it: rapid review of the evidence. Lancet..

[6] https://www.bma.org.uk/news-and-opinion/no-time-to-be-a-student. Last accessed 15 Feb 2021

[7] Lai J, Ma S, Wang Y, Cai Z, Hu J, Wei N (2020). Factors Associated With Mental Health Outcomes Among Health Care Workers Exposed to Coronavirus Disease 2019. JAMA Netw Open..

[8] Smith CA (2020). Covid-19: healthcare students face unique mental health challenges. BMJ..

[9] Gallè F, Sabella EA, Da Molin G, De Giglio O, Caggiano G, Di Onofrio V, Ferracuti S, Montagna MT, Liguori G, Orsi GB, Napoli C (2020). Understanding Knowledge and Behaviors Related to CoViD-19 Epidemic in Italian Undergraduate Students: The EPICO Study. Int J Environ Res Public Health..

[10] Saraswathi I, Saikarthik J, Senthil Kumar K, Madhan Srinivasan K, Ardhanaari M, Gunapriya R (2020). Impact of COVID-19 outbreak on the mental health status of undergraduate medical students in a COVID-19 treating medical college: a prospective longitudinal study. PeerJ..

[11] Ye W, Ye X, Liu Y, Liu Q, Vafaei S, Gao Y, Yu H, Zhong Y, Zhan C (2020). Effect of the Novel Coronavirus Pneumonia Pandemic on Medical Students' Psychological Stress and Its Influencing Factors. Front Psychol..

[12] Nakhostin-Ansari A, Sherafati A, Aghajani F, Khonji MS, Aghajani R, Shahmansouri N (2020). Depression and Anxiety among Iranian Medical Students during COVID-19 Pandemic. Iran J Psychiatry..

[13] Wang QQ, Fang YY, Huang HL, Lv WJ, Wang XX, Yang TT, et al. Anxiety, depression and cognitive emotion regulation strategies in Chinese nurses during the COVID-19 outbreak. J Nurs Manag. 2021; Jan 22 (Epub ahead of print). 10.1111/jonm.13265 PMC8013387 33480056

[14] Saddik B, Hussein A, Sharif-Askari FS, Kheder W, Temsah MH, Koutaich RA (2020). Increased Levels of Anxiety Among Medical and Non-Medical University Students During the COVID-19 Pandemic in the United Arab Emirates. Risk Manag Healthc Policy..

[15] https://www.ons.gov.uk/methodology/classificationsandstandards/measuringequality/ethnicgroupnationalidentityandreligion. Last accessed 15 Feb 2021

[16] Rotenstein LS, Ramos MA, Torre M, Segal JB, Peluso MJ, Guille C (2016). Prevalence of Depression, Depressive Symptoms, and Suicidal Ideation Among Medical Students: A Systematic Review and Meta-Analysis. JAMA..

[17] O'Byrne L, Gavin B, McNicholas F (2020). Medical students and COVID-19: the need for pandemic preparedness. J Med Ethics..

[18] Representatives of the STARSurg Collaborative, EuroSurg Collaborative, and TASMAN Collaborative. Medical student involvement in the COVID-19 response. Lancet. 2020;395(10232):1254. 10.1016/S0140-6736(20)30795-9 PMC7270863 32247322

[19] Hanbazaza M, Wazzan H (2021). Changes in eating habits and lifestyle during COVID-19 curfew in children in Saudi Arabia. Nutr Res Pract..

[20] Lewis R, Roden LC, Scheuermaier K, Gomez-Olive FX, Rae DE, Iacovides S (2021). The impact of sleep, physical activity and sedentary behaviour on symptoms of depression and anxiety before and during the COVID-19 pandemic in a sample of South African participants. Sci Rep..

[21] Aghakhani K, Shalbafan M (2020). What COVID-19 outbreak in Iran teaches us about virtual medical education. Med Educ Online..

[22] https://www.theguardian.com/education/2020/mar/22/coronavirus-forces-medical-students-sit-final-exams-online. Last accessed 15 Feb 2021

[23] Pettitt-Schieber B, Kuo M, Steehler A, Dong A, Fakunle O, Manalo T (2021). Implementation and evaluation of eight virtual surgical electives for medical students during the COVID-19 pandemic. Am J Surg..

[24] Sharma D, Bhaskar S (2020). Addressing the Covid-19 Burden on Medical Education and Training: The Role of Telemedicine and Tele-Education During and Beyond the Pandemic. Front Public Health..

[25] Lalloo F, Hawkins N, Lindley R, Kumar S. Medical students as service learners: opportunities, risks and recommendations. Educ. Prim Care. 2021:1–5. 10.1080/14739879.2020.1869589 33586625

[26] Salamonson Y, Everett B, Koch J, Wilson I, Davidson PM (2009). Learning strategies of first year nursing and medical students: a comparative study. Int J Nurs Stud..

[27] Bengtsson M, Ohlsson B (2010). The nursing and medical students motivation to attain knowledge. Nurse Educ Today..

[28] Dyrbye LN, Eacker A, Durning SJ, Brazeau C, Moutier C, Massie FS, Satele D, Sloan JA, Shanafelt TD (2015). The Impact of Stigma and Personal Experiences on the Help-Seeking Behaviors of Medical Students With Burnout. Acad Med..

