# Supplementary material for: The impact of the COVID-19 pandemic on the lifestyle and behaviours, mental health and education of students studying healthcare-related courses at a British university
Source: BMC Med Educ. 2022 Feb 21;22:115. doi: 10.1186/s12909-022-03179-z (PMC8860134; doi:10.1186/s12909-022-03179-z)
Supplement: Supplementary file 2 — Additional file 2. [file 12909_2022_3179_MOESM2_ESM.pdf]

The impact of COVID-19 on the mental health and education of students at a British University.

Showing 233 of 233 responses

Showing **all** responses

Showing **all** questions

Response rate: 233%

1 What course do you study?

Showing all 232 responses	
Medicine	611710-611701-63868315
Medicine MbChB	611710-611701-64904945
Medicine	611710-611701-64905108
Medicine	611710-611701-64905508
Medicine	611710-611701-64905997
Medicine	611710-611701-64906133
Medicine	611710-611701-64906117
Medicine	611710-611701-64906786
Medicine	611710-611701-64908178
Medicine	611710-611701-64908956
Medicine	611710-611701-64910550
Medicine	611710-611701-64911954
Medicine	611710-611701-64912202
Medicine	611710-611701-64912999
Medicine	611710-611701-64913058
Medicine	611710-611701-64913721
Medicine	611710-611701-64914840
Medicine	611710-611701-64917154
Medicine	611710-611701-64921771
Medicine	611710-611701-64924303
Medicine	611710-611701-64928259

Medicine	611710-611701-64975966
Medicine	611710-611701-65180318
Medicine	611710-611701-65180949
Medicine	611710-611701-65238328
Medicine	611710-611701-65238969
Paramedic Science	611710-611701-65339701
Paramedic science	611710-611701-65339700
MRI MSc	611710-611701-65339699
Paramedic studies	611710-611701-65339690
Medical Science	611710-611701-65339766
Paramedic science	611710-611701-65339840
Medical science	611710-611701-65339703
BSc Paramedic Science	611710-611701-65339825
Msc healthcare management	611710-611701-65339818
Medical science	611710-611701-65339708
BSc (Hons) Paramedic Science	611710-611701-65339861
Operating department practitioner	611710-611701-65339886
MSc Healthcare Management	611710-611701-65339765
Operating department Practitioner	611710-611701-65339824
MSc Pharmaceutical Sciences	611710-611701-65339804
Medical Science	611710-611701-65339712
Medical Science BSc	611710-611701-65339893
Nutritional science	611710-611701-65340223
MBA healthcare management	611710-611701-65340228
MBA healthcare Management	611710-611701-65339978
Paramedic science	611710-611701-65339809
Public Health	611710-611701-65340293
Medical Science	611710-611701-65340410
Pharmaceutical science	611710-611701-65340365
Paramedic Science	611710-611701-65340650
Operating Department Practice	611710-611701-65340388
paramedic science	611710-611701-65340847
Paramedic science (BSc)	611710-611701-65339855

Medical Science	611710-611701-65340923
Operating department practitioner	611710-611701-65339954
Paramedic Science	611710-611701-65340939
Medical Science	611710-611701-65340898
Operating department practice	611710-611701-65340848
BSc (Hons) Medical Science	611710-611701-65341347
Paramedic	611710-611701-65341388
Msc MRI	611710-611701-65341475
Paramedic science	611710-611701-65341123
Paramedic science	611710-611701-65342091
ODP	611710-611701-65341995
BSc paramedic science	611710-611701-65339772
Paramedic Science	611710-611701-65342358
Medical science	611710-611701-65342802
Medical science	611710-611701-65343006
Pharmaceutical Science	611710-611701-65339702
MSc Healthcare Management	611710-611701-65343532
Medical science	611710-611701-65344014
MSC Healthcare Management	611710-611701-65344745
medical science with foundation year	611710-611701-65344919
Physician Associate MCs	611710-611701-65346039
Medical science	611710-611701-65346751
paramedic science	611710-611701-65347433
Paramedic Science	611710-611701-65347985
Paramedic science	611710-611701-65348095
Paramedic Science	611710-611701-65348420
BSc Paramedic Science	611710-611701-65348617
Paramedical science	611710-611701-65348729
Public Health	611710-611701-65350542
Paramedic Science	611710-611701-65351175
Bsc Paramedic Science	611710-611701-65351672
MSc public health	611710-611701-65351589
Paramedic science	611710-611701-65353054

Paramedic science	611710-611701-65353753
Bsc management and leadership in health and social care	611710-611701-65353859
Healthcare management mba	611710-611701-65355362
Paramedic Science	611710-611701-65355666
Dip Ed paramedic	611710-611701-65351657
Paramedic Science	611710-611701-65356103
Operating Department Practice	611710-611701-65357763
Paramedic Science	611710-611701-65358813
Msc public health	611710-611701-65358699
Paramedic science	611710-611701-65358950
Medicine	611710-611701-65360204
Medicine	611710-611701-65360786
BSc (Hons) Paramedic Science	611710-611701-65362055
Medicine	611710-611701-65363079
Paramedic Science	611710-611701-65363373
Msc public health	611710-611701-65363646
Public health MSc	611710-611701-65364335
Medical science	611710-611701-65365724
BSc Public health	611710-611701-65366876
Medical science	611710-611701-65367249
MSc. Physician Associate	611710-611701-65367775
Physician Associate Studies	611710-611701-65369259
Medical science	611710-611701-65375617
operating department praction	611710-611701-65376195
Paramedic science	611710-611701-65377486
ODP	611710-611701-65380858
Paramedic science	611710-611701-65382930
Pharmaceutical science	611710-611701-65394778
BSc(Hons) Public Health	611710-611701-65394930
Public health	611710-611701-65396871
Paramedic Science	611710-611701-65404525
Paramedic science	611710-611701-65407022
ODP	611710-611701-65410678

medical science	611710-611701-65417826
MSc Physician Associate	611710-611701-65420112
Public health	611710-611701-65423303
MSc public health	611710-611701-65430088
ODP	611710-611701-65434612
Bsc (Hons) Medical science	611710-611701-65440867
masters in public health	611710-611701-65441962
mental health nursing	611710-611701-65443108
MSc Healthcare Management	611710-611701-65444987
Medical science	611710-611701-65447207
Pharmaceutical science	611710-611701-65447865
BSc Public Health	611710-611701-65450534
BsC honours medical science	611710-611701-65453185
Paramedic Science	611710-611701-65471280
ODP	611710-611701-65471517
Biomedical science	611710-611701-65483704
odp	611710-611701-65484370
Pharmaceutical science	611710-611701-65512965
PhD	611710-611701-65543617
medicine	611710-611701-65559487
Operating Department Practice	611710-611701-65571031
PhD Medical Science	611710-611701-65579375
Medicine	611710-611701-65580622
BSc Medical Science	611710-611701-65582736
Adult Nursing	611710-611701-65590713
Operating department practice	611710-611701-65582499
Medicine	611710-611701-65605735
Leadership in health and social care	611710-611701-65605489
Physician Associate	611710-611701-65618375
Nursing	611710-611701-65632523
Paramedic Science	611710-611701-65647057
Medicine	611710-611701-65656449
Medicine	611710-611701-65657915

Medicine	[illegible]
adult nursing	611710-611701-65677803
Medical Science	611710-611701-65680201
MENTAL HEALTH	611710-611701-65684000
Public Health	611710-611701-65689916
MA Social Work	611710-611701-65693396
EdD	611710-611701-65695895
Medicine	611710-611701-65697993
Medicine	611710-611701-65727947
BA Social Work	611710-611701-65744048
BSc(HONS)international Business management	611710-611701-65754244
Msc public health	611710-611701-65757495
child nursing	611710-611701-65757957
Medicine	611710-611701-65776398
Operation Department Practitioner	611710-611701-65797593
Adult nursing	611710-611701-65823469
MA Early Childhood Education	611710-611701-65833592
ODP	611710-611701-65838732
Business Management	611710-611701-65880395
MSc in HealthCare Management	611710-611701-65895037
Early Childhood Studies	611710-611701-65947725
Medicine	611710-611701-65952652
Medicine	611710-611701-65952660
medicine	611710-611701-65952716
Medicine	611710-611701-65952826
Medicine	611710-611701-65952856
Medicine	611710-611701-65952944
Medicine	611710-611701-65953035
Medicine	611710-611701-65953635
Medicine	611710-611701-65954555
Medicine	611710-611701-65955274
Medicine	611710-611701-65959417
PhD Health and Social Care	611710-611701-65968379
BSc. Nursing (Adult)	[illegible]

BSc. Nursing (Adult)	611710-611701-65972122
Adult Nurse	611710-611701-66017384
Diploma in Paramedic Science	611710-611701-66022412
Social Work BA	611710-611701-66034596
BA Early Childhood Studies	611710-611701-66040870
Medicine	611710-611701-66163273
PhD	611710-611701-66240947
MSc public health	611710-611701-66315099
Adult nursing	611710-611701-66400566
Master's in Public Health	611710-611701-66473167
Midwifery	611710-611701-66730747
Midwifery	611710-611701-66731179
Midwifery	611710-611701-66731323
Midwifery	611710-611701-66731367
Midwifery	611710-611701-66731385
Midwifery	611710-611701-66731500
Midwifery	611710-611701-66731616
Midwifery	611710-611701-66731604
Midwifery	611710-611701-66733611
Primary education studies	611710-611701-66737100
Midwifery	611710-611701-66738777
Bsc midwifery	611710-611701-66739149
Midwifery	611710-611701-66750717
Primary Education	611710-611701-66753644
Child Nursing	611710-611701-66766928
Paramedic science	611710-611701-66832802
Paediatric Nursing	611710-611701-66894318
Mental Health Nursing	611710-611701-66894651
Adult Nursing	611710-611701-66906613
Operating Department Practice	611710-611701-66936452
mental health nursing	611710-611701-66947932
midwifery	611710-611701-66948498
Adult nursing	611710-611701-66978483

Social Work	611710-611701-67017947
social work	611710-611701-67025772
Medicine	611710-611701-67046848
Medicine	611710-611701-67046840
BA Social Work	611710-611701-67064075
Adult Nursing	611710-611701-67082205
BSc Adult Nursinf	611710-611701-67084380
Adult Nursing	611710-611701-67084975
Nursing	611710-611701-67090483
Medicine	611710-611701-67189021
Mental nursing	611710-611701-67253342
Paramedic Science	611710-611701-67254500
mental health nursing	611710-611701-67272372
Mental health nursing	611710-611701-67274548

2 What year of study are you in/ will you be in the academic year 2020/2021?

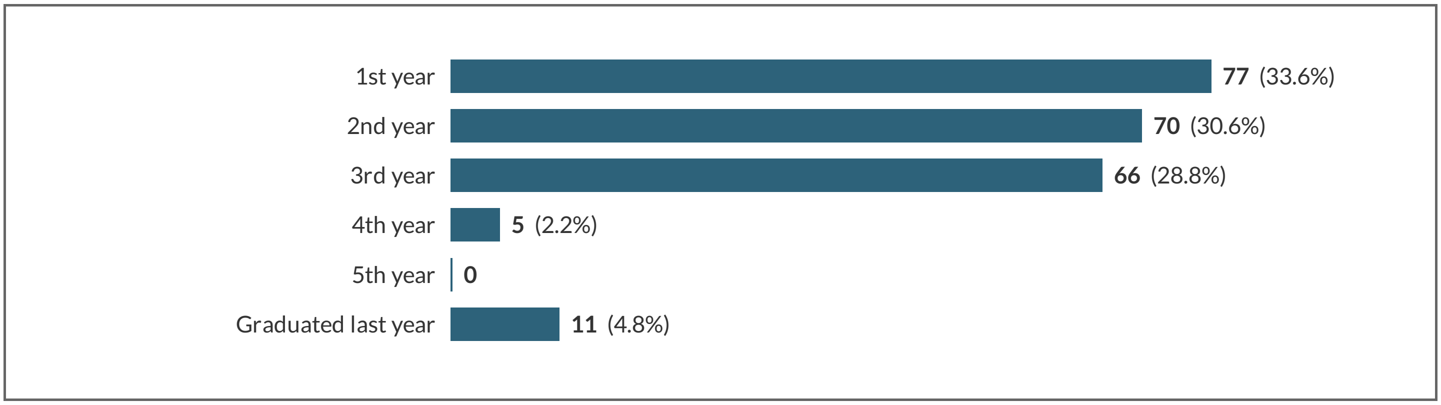

3 Gender?

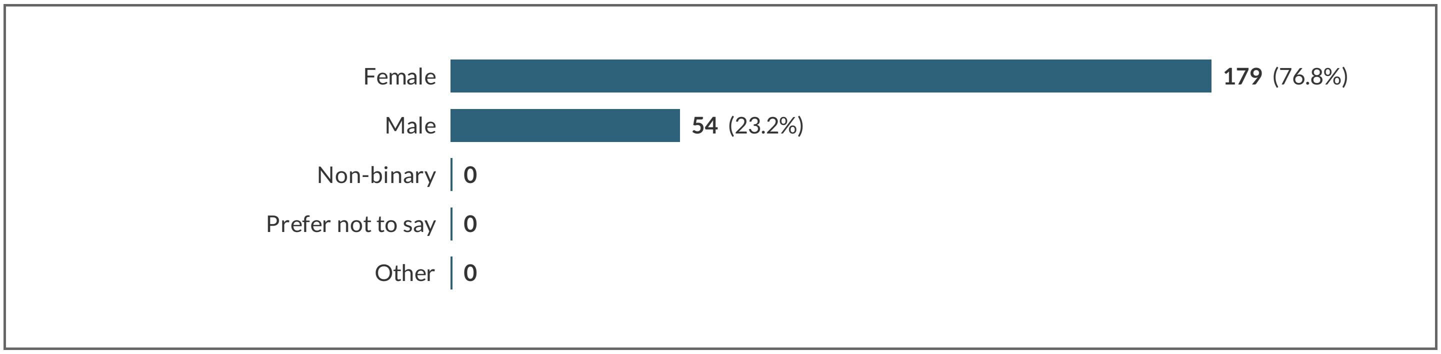

3.a If you selected Other, please specify how you prefer to self-describe:

No responses

4 Age?

Showing all 233 responses	
23	611710-611701-63868315
23	611710-611701-64904945
20	611710-611701-64905108
21	611710-611701-64905508
20	611710-611701-64905997
19	611710-611701-64906133
20	611710-611701-64906117
21	611710-611701-64906786
21	611710-611701-64908178
20	611710-611701-64908956
26	611710-611701-64910550
23	611710-611701-64911954
20	611710-611701-64912202
23	611710-611701-64912999
24	611710-611701-64913058
21	611710-611701-64913721
26	611710-611701-64914840
20	611710-611701-64917154
26	611710-611701-64921771
20	611710-611701-64924303
20	611710-611701-64928259
20	611710-611701-64975966
20	611710-611701-65180318
19	611710-611701-65180949
19	611710-611701-65238328
23	611710-611701-65238969
20	611710-611701-65339701

19	611710-611701-65339700
34	611710-611701-65339699
37	611710-611701-65339690
22	611710-611701-65339766
21	611710-611701-65339840
21	611710-611701-65339703
27	611710-611701-65339825
33	611710-611701-65339818
40	611710-611701-65339708
22	611710-611701-65339861
18	611710-611701-65339886
44	611710-611701-65339765
25	611710-611701-65339824
23	611710-611701-65339804
21	611710-611701-65339712
34	611710-611701-65339893
25	611710-611701-65340223
25	611710-611701-65340228
25	611710-611701-65339978
21	611710-611701-65339809
21	611710-611701-65340293
20	611710-611701-65340410
19	611710-611701-65340365
20	611710-611701-65340650
54	611710-611701-65340388
20	611710-611701-65340847
20	611710-611701-65339855
21	611710-611701-65340923
27	611710-611701-65339954
21	611710-611701-65340939
25	611710-611701-65340898
33	611710-611701-65340848
20	611710-611701-65341347

42	611710-611701-65341388
34	611710-611701-65341475
35	611710-611701-65341123
21	611710-611701-65342091
28	611710-611701-65341995
19	611710-611701-65339772
21	611710-611701-65342358
27	611710-611701-65342802
18	611710-611701-65343006
23	611710-611701-65339702
43	611710-611701-65343532
20	611710-611701-65344014
40	611710-611701-65344745
17	611710-611701-65344919
36	611710-611701-65346039
57	611710-611701-65346751
22	611710-611701-65347433
20	611710-611701-65347985
19	611710-611701-65348095
31	611710-611701-65348420
22	611710-611701-65348617
21	611710-611701-65348729
19	611710-611701-65350542
21	611710-611701-65351175
19	611710-611701-65351672
25	611710-611701-65351589
22	611710-611701-65353054
20	611710-611701-65353753
28	611710-611701-65353859
32	611710-611701-65355362
20	611710-611701-65355666
42	611710-611701-65351657
20	611710-611701-65356103

[illegible]	[illegible]
22	611710-611701-65357763
19	611710-611701-65358813
26	611710-611701-65358699
34	611710-611701-65358950
20	611710-611701-65360204
19	611710-611701-65360786
21	611710-611701-65362055
20	611710-611701-65363079
23	611710-611701-65363373
25	611710-611701-65363646
46	611710-611701-65364335
43 años	611710-611701-65365724
20	611710-611701-65366876
20	611710-611701-65367249
35	611710-611701-65367775
22	611710-611701-65369259
18	611710-611701-65375617
21	611710-611701-65376195
30	611710-611701-65377486
19	611710-611701-65380858
21	611710-611701-65382930
20	611710-611701-65394778
43	611710-611701-65394930
22	611710-611701-65396871
29	611710-611701-65404525
41	611710-611701-65407022
39	611710-611701-65410678
20	611710-611701-65417826
22	611710-611701-65420112
31	611710-611701-65423303
59	611710-611701-65430088
21	611710-611701-65434612
[illegible]	[illegible]

18	611710-611701-65440867
28	611710-611701-65441962
46	611710-611701-65443108
39	611710-611701-65444987
33	611710-611701-65447207
21	611710-611701-65447865
50	611710-611701-65450534
18	611710-611701-65453185
21	611710-611701-65471280
38	611710-611701-65471517
25	611710-611701-65483704
38	611710-611701-65484370
22	611710-611701-65512965
24	611710-611701-65543617
20	611710-611701-65559487
54	611710-611701-65571031
24	611710-611701-65579375
20	611710-611701-65580622
21	611710-611701-65582736
47	611710-611701-65590713
28	611710-611701-65582499
20	611710-611701-65605735
32	611710-611701-65605489
46	611710-611701-65618375
27	611710-611701-65632523
24	611710-611701-65647057
20	611710-611701-65656449
19	611710-611701-65657915
37	611710-611701-65677803
19	611710-611701-65680201
30-40	611710-611701-65684000
21	611710-611701-65689916
38	611710-611701-65693396

62	611710-611701-65695895
20	611710-611701-65697993
20	611710-611701-65727947
47	611710-611701-65744048
45	611710-611701-65754244
27	611710-611701-65757495
18	611710-611701-65757957
28	611710-611701-65776398
59	611710-611701-65797593
19	611710-611701-65823469
26	611710-611701-65833592
20	611710-611701-65838732
27	611710-611701-65880395
41	611710-611701-65895037
43	611710-611701-65947725
18	611710-611701-65952652
19	611710-611701-65952660
19	611710-611701-65952716
18	611710-611701-65952826
20	611710-611701-65952856
21	611710-611701-65952944
18	611710-611701-65953035
19	611710-611701-65953635
18	611710-611701-65954555
18	611710-611701-65955274
23	611710-611701-65959417
30	611710-611701-65968379
39	611710-611701-65972122
23	611710-611701-66017384
50	611710-611701-66022412
35	611710-611701-66034596
40	611710-611701-66040870
21	611710-611701-66163273

54	611710-611701-66180510
37	611710-611701-66240947
30	611710-611701-66315099
18	611710-611701-66400566
25	611710-611701-66473167
18	611710-611701-66730747
28	611710-611701-66731179
29	611710-611701-66731323
18	611710-611701-66731367
18	611710-611701-66731385
20	611710-611701-66731500
21	611710-611701-66731616
19	611710-611701-66731604
18	611710-611701-66733611
20	611710-611701-66737100
22	611710-611701-66738777
28	611710-611701-66739149
18	611710-611701-66750717
24	611710-611701-66753644
31	611710-611701-66766928
21	611710-611701-66832802
37	611710-611701-66894318
39	611710-611701-66894651
28	611710-611701-66906613
43	611710-611701-66936452
32	611710-611701-66947932
31	611710-611701-66948498
40	611710-611701-66978483
20	611710-611701-67017947
22	611710-611701-67025772
19	611710-611701-67046848
23	611710-611701-67046840
34	611710-611701-67064075

24	611710-611701-67082205
19	611710-611701-67084380
25	611710-611701-67084975
19	611710-611701-67090483
19	611710-611701-67189021
31	611710-611701-67253342
39	611710-611701-67254500
32	611710-611701-67272372
44	611710-611701-67274548

5 Ethnicity?

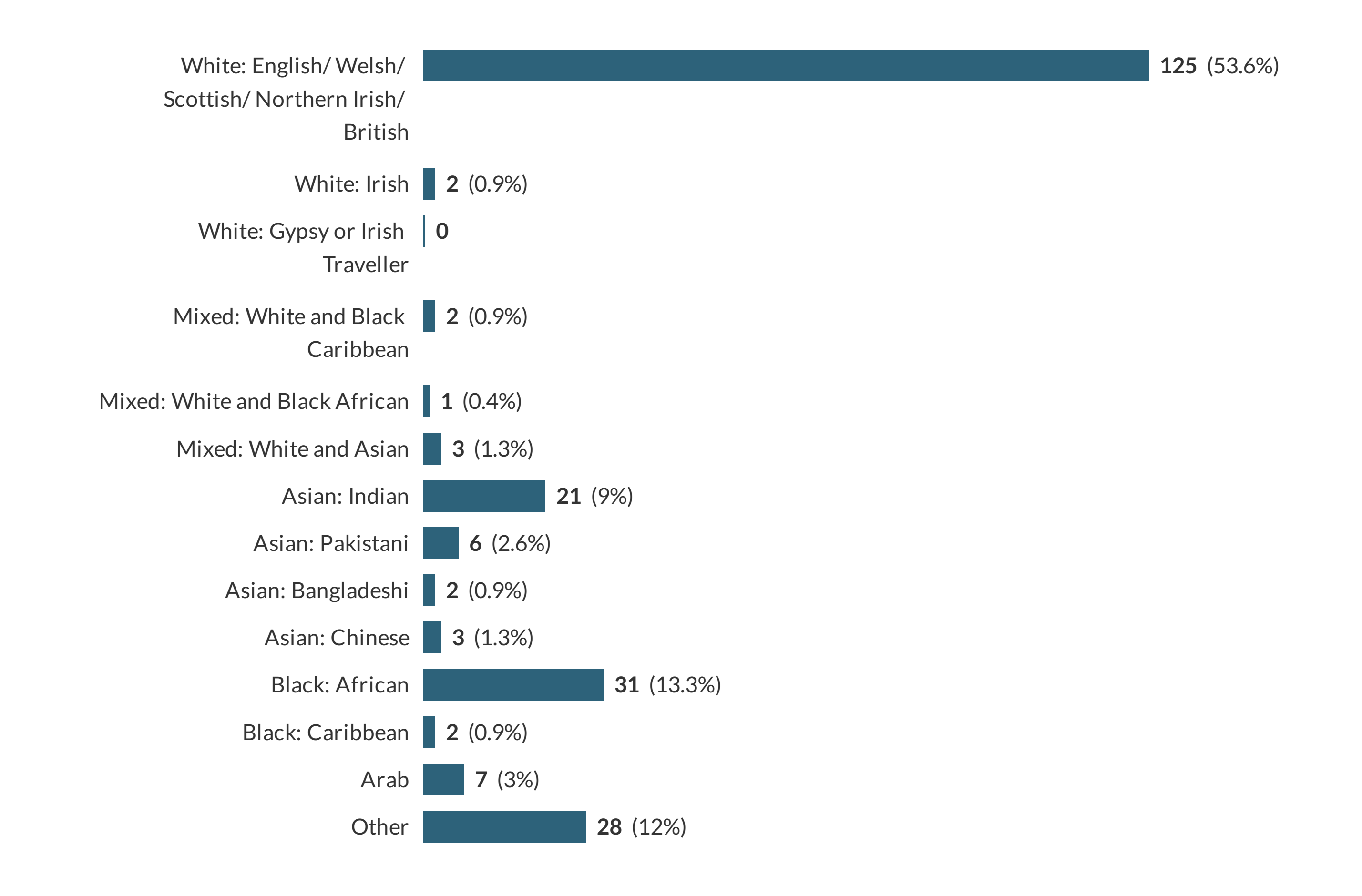

5.a If you selected Other, please specify your ethnic group:

Showing all 28 responses	
White European	611710-611701-65339703
Mixed: White	611710-611701-65339712
White: Portuguese	611710-611701-65340365
White British American	611710-611701-65340650
White - Portuguese	611710-611701-65340898
Tamil (Sri Lanka)	611710-611701-65341347
Filipino	611710-611701-65341995
Mixed afro-euroasian	611710-611701-65342358
White Bulgarian	611710-611701-65343532
White other	611710-611701-65346039
Afghan	611710-611701-65348729
White	611710-611701-65353054
White: Russian	611710-611701-65376195
Asian: Thai	611710-611701-65380858
White other	611710-611701-65407022
Nepalese	611710-611701-65420112
White	611710-611701-65483704
Asian - pakistani/turkish	611710-611701-65559487
Western Europe	611710-611701-65590713
white european	611710-611701-65605489
White European	611710-611701-65618375
polish	611710-611701-65677803
African	611710-611701-65797593
kurdish	611710-611701-65838732
White other	611710-611701-65880395
Sri Lankan	611710-611701-65952652
Other Asian background	611710-611701-65972122
Other asian	611710-611701-66739149

6 Religion?

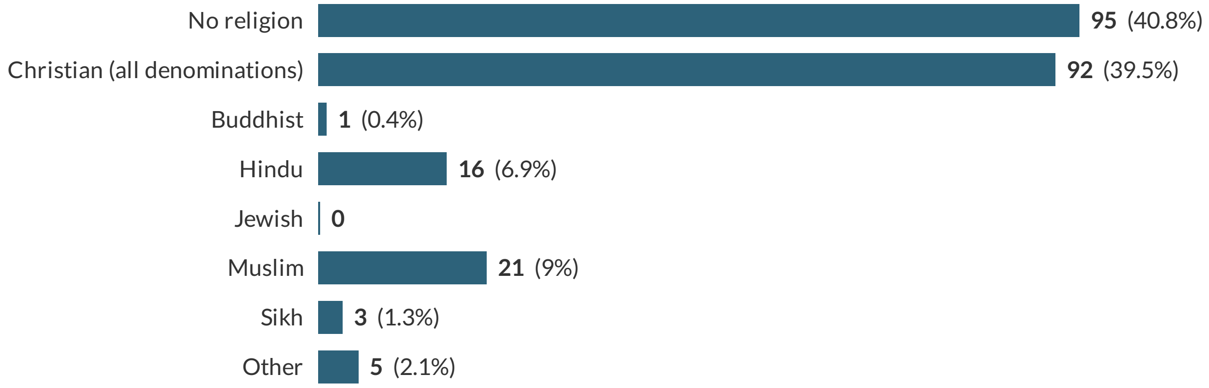

6.a If you selected Other, please specify your religion:

Showing all 5 responses	
Agnostic	611710-611701-65396871
Pagan	611710-611701-65434612
Jain	611710-611701-65697993
Catholic Christian	611710-611701-65797593
Pagan	611710-611701-67254500

7 EDUCATION: please choose one answer for each of the following questions. (important note: row 4 should say "your course" rather than specifically "medical school". Students studying any course can answer this question.)

7.1 1. At the Beginning of the lockdown period, you were provided with all the information needed to continue with your academic studies.

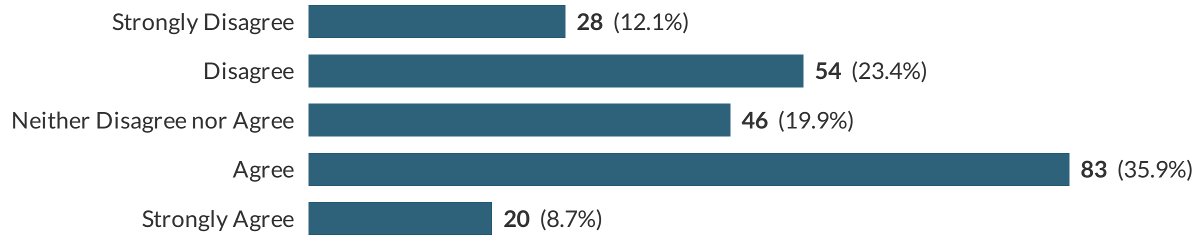

Multi answer: Percentage of respondents who selected each answer option (e.g. 100% would represent that all this question's respondents chose that option)

7.2 2. Sometimes you struggle to complete the learning outcomes in the context of the online delivery

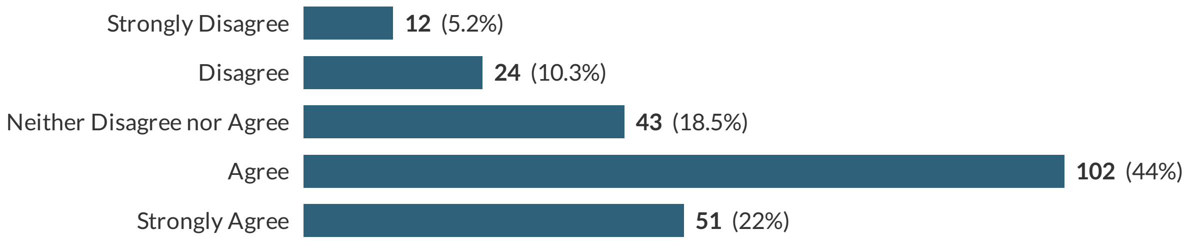

Multi answer: Percentage of respondents who selected each answer option (e.g. 100% would represent that all this question's respondents chose that option)

7.3 3. You are worried that your practical skills (e.g. clinical skills) will be affected.

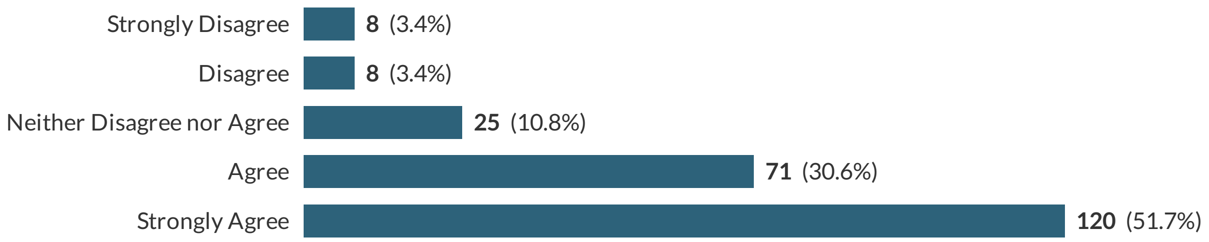

Multi answer: Percentage of respondents who selected each answer option (e.g. 100% would represent that all this question's respondents chose that option)

7.4 4. Since February 2020 (the start of the pandemic) you have considered leaving medical school.

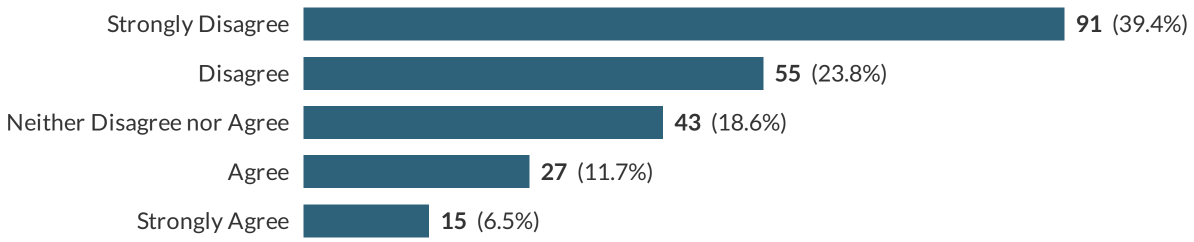

Multi answer: Percentage of respondents who selected each answer option (e.g. 100% would represent that all this question's respondents chose that option)

7.5 5. You are worried about the impact of COVID-19 might have on your future career.

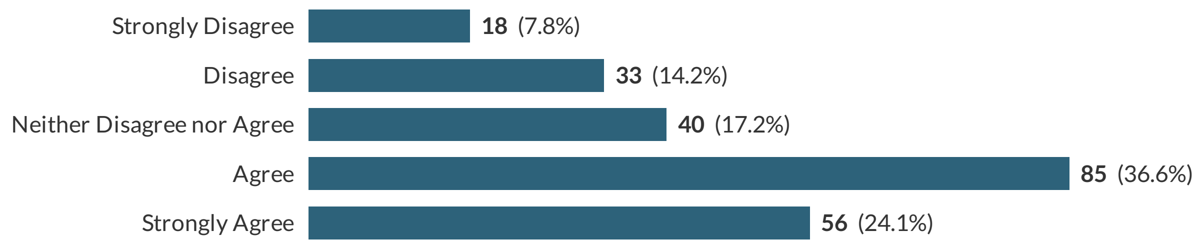

Multi answer: Percentage of respondents who selected each answer option (e.g. 100% would represent that all this question's respondents chose that option)

7.6 6. Your academic studies have been adequately supported since the start of the pandemic.

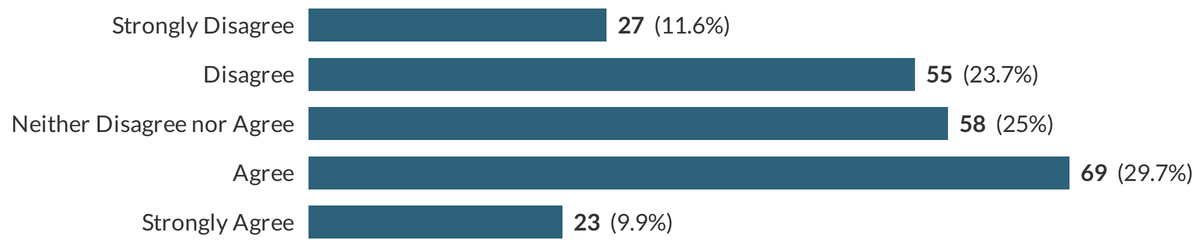

Multi answer: Percentage of respondents who selected each answer option (e.g. 100% would represent that all this question's respondents chose that option)

7.7 7. Online teaching and resources provided by the university has made up for cancelled face-to-face teaching, including placements.

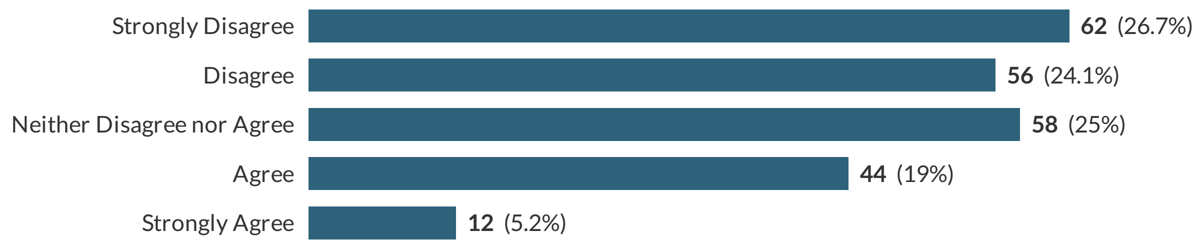

Multi answer: Percentage of respondents who selected each answer option (e.g. 100% would represent that all this question's respondents chose that option)

7.8 8. Lockdown online teaching compares well to conventional lectures prior to lockdown.

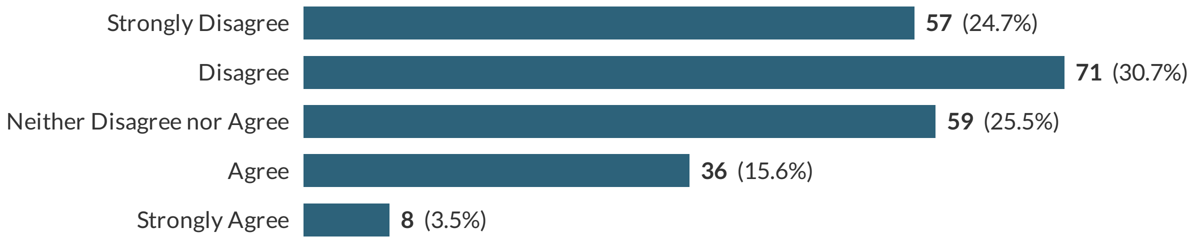

Multi answer: Percentage of respondents who selected each answer option (e.g. 100% would represent that all this question's respondents chose that option)

7.9 9. Online and virtual teaching and resources should be part of the standard curriculum even after lockdown ends.

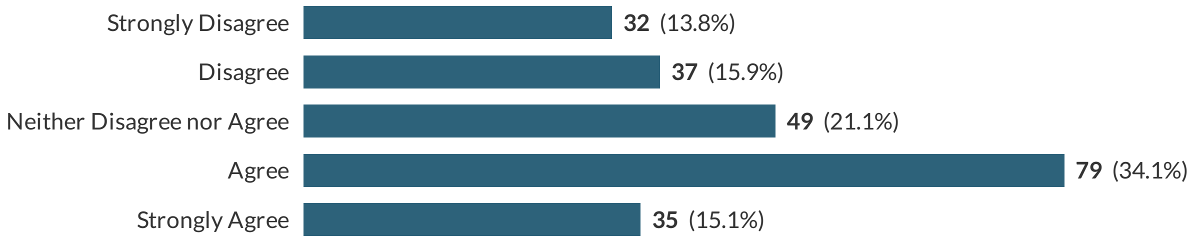

Multi answer: Percentage of respondents who selected each answer option (e.g. 100% would represent that all this question's respondents chose that option)

---

8 Any other comments regarding medical education:

**Showing all 42 responses**	
Although online teaching went well considering the circumstances, I felt it lacked proper structure	611710-611701-63868315
Access to resources such as OSMOSIS etc was very helpful!	611710-611701-64905508
There is too much practical session that I have missed out on that will not be made up for later.	611710-611701-64906133
	611710-611701-64908178
I found the zoom lectures very effective. Especially as I was able to rewatch the recordings of some subject I found more challenging. I have missed the clinical skills and anatomy lessons terribly and am worried about the impact of this, especially the clinical skills as there simply is no other way to practice these effectively.	611710-611701-64910550
None.	611710-611701-64913721
Registered medical staff shouldn't have to do a PGCert to be able to teach full time and pass on medical knowledge as a lecturer	611710-611701-64921771
[illegible]	[illegible]

So far I like how Year 3 is being delivered but in year 2 at the start of the lockdown they were veryyy late to give us the online lectures and resources we needed, they ended up having revision lectures a few days before our exams which left us very little time to revise! And I still don’t understand Renal block, few people do	611710-611701-65238969
I feel it is too early to ask these questions. My course hasn't started yet. My course ended in April and it was very new and scary for everyone then. I feel like communication could have been better , but haven't sampled any of the new online strategies yet. I am currently anxious about the upcoming year . Maybe ask these questions again in December ? I feel I'd be able to give a clearer answer	611710-611701-65339708
My course is non fave to face anyway. It is all online via distance learning.	611710-611701-65339765
The classroom provides a distraction free peer supported environment of learning that I don’t believe can be replicated via online learning.	611710-611701-65340293
Learning Clinical skills on pla cement is really important and one of the main reasons I chose the course. Concerned I won’t get enough experience during first year.	611710-611701-65340388
Didn't receive proper online lectures. Just given Q+As and a small introduction each week about 10-30 minutes. Although we have fed this back to the course this week and they responded that they will change this and had been doing this for flexibility of the students, understandably. They apologised and said they will change it. So this issue is hopefully being sorted for future learning.	611710-611701-65339855
Online learning cannot replace the valuable lessons learnt on clinical placement.	611710-611701-65340939
Online teaching resources have been adequate but can't in any way make up for the practical placement I missed out on. I also feel there has been a lack of guidance on returning to placement and what may happen in the future with placement for students if there is a second wave.	611710-611701-65340848
I don't go to medical school	611710-611701-65341347
The online teaching was extremely poor. But so was the delivery before lockdown.	611710-611701-65341123
The university should have just continued in September as not all student work well at home	611710-611701-65339702
I like the zoom lectures. I am concerned how this years delivery of work will work out for me as I’m a visual learner.	611710-611701-65346751
Please get us back full time like schools have done.	611710-611701-65348617
Research studies was very difficult for most students doing MBA healthcare management. There were no support and the lecturer not helpful at all. Most people failed the course due to not enough information.	611710-611701-65355362
It is hard to comment on the online resources as I have only had one online lecture back in May. My year did not receive online teaching throughout the rest of lockdown.	611710-611701-65355666
Working from home is ideal due to travel distance to university. But online lessons shockingly bad no direction, online library difficult to use no directions from university staff poor exam marking, poor Direction on	611710-611701-65351657

directions from university staff poor exam marking, poor Direction on learning outcomes difficult to get support, disability SRA ignored.	
I have no comments to write .	611710-611701-65363646
stuff should consider students when marking their work because Covid-19 have had effect on study. Marking scheme should be lowered	611710-611701-65364335
Online lectures has been beneficial for me as a mature student living at home	611710-611701-65382930
We received no support, very little teaching or communication and were left to try to learn what we needed to know with no guidance. As a result many of the class failed elements of the course and have to retest causing stress and anxiety as well as preventing us from progressing in our careers	611710-611701-65407022
No	611710-611701-65423303
N/a	611710-611701-65471517
online teaching is definitely not a substitute to face-to-face teaching, but some aspects could be transitioned to online based easily.	611710-611701-65559487
All changes have not been necessarily bad	611710-611701-65656449
WHEN IS THIS PANDEMIC ENDING?	611710-611701-65684000
No	611710-611701-65754244
An a PhD student, I have mainly accessed supervisions online rather than training. I have access an online research conference and several training sessions. The support from my supervisors has been excellent. The support from the wider doctoral school has been average.	611710-611701-66240947
I feel like we have been left alone to self teach with only a few Q&A sessions with our lecturers. I expected more online lectures.	611710-611701-66738777
I have found it hard learning online as I am a visual learner, but it is useful to have the online resources to go back too	611710-611701-66766928
Although I only started september so have no basis for comparison in ARU, i have attended university and gained an undergrad degree previously and ARU's standards are much higher, despite covid affecting education delivery. I have felt far more supported, far more educated and feel more on top of my studies than i did in my prior 3 year degree. This has also made childcare a lot easier as I do not have added commute time.	611710-611701-66948498
none	611710-611701-66978483
Powerpoints etc should be more closely monitored by faculty	611710-611701-67046840
It is difficult to make a comparison with face to face teaching as I have not experienced that yet. I think the main thing I am missing out on is interaction with my peers and teachers	611710-611701-67064075
Personal tutor should really help new student for at least for 4 weeks, MS teams should be use more frequently for lectures not recorded video for student with learning disability and also there student who are not outspoken, it does not mean they don't have a problem, hence personal tutor should do a 1:1 within that first 4 weeks of uni. also a follow email to check on our welfare and asked how we are doing once every term.	611710-611701-67253342

no	611710-611701-67272372

9 Since the start of the pandemic:

9.1 10. Has your diet changed?

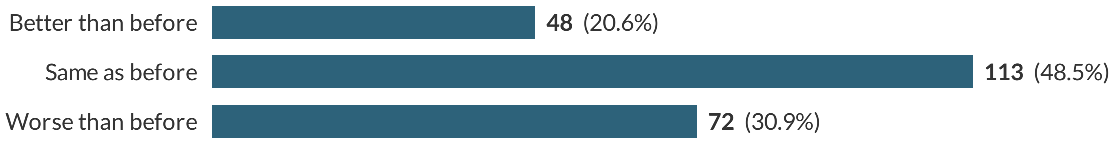

Multi answer: Percentage of respondents who selected each answer option (e.g. 100% would represent that all this question's respondents chose that option)

10 Since the start of the pandemic:

10.1 11. Do you now smoke?

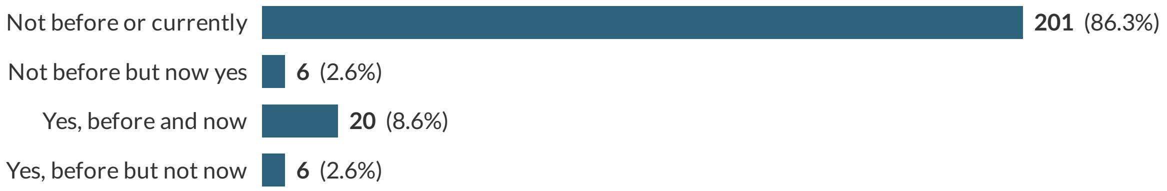

Multi answer: Percentage of respondents who selected each answer option (e.g. 100% would represent that all this question's respondents chose that option)

11 Since the start of the pandemic:

11.1 12. Has your level of physical activity changed?

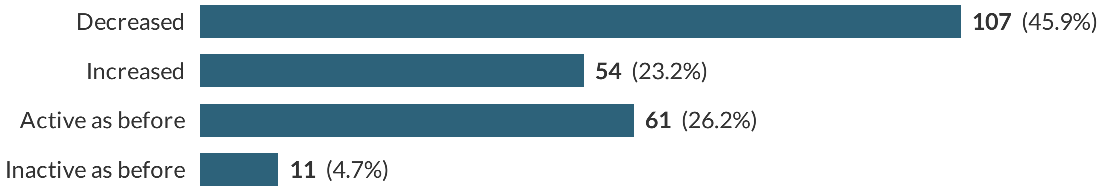

Multi answer: Percentage of respondents who selected each answer option (e.g. 100% would represent that all this question's respondents chose that option)

12 Any other comments regarding lifestyle & habits:

Showing all 34 responses	
Sleep routine is worse now	611710-611701-64905508
I go to the gym less but I have a physically demanding day job	611710-611701-64910550
None.	611710-611701-64913721
Due to not wanting to share the train carriages with others I have begun cycling (15 miles) to get to my part time job	611710-611701-64921771
I go to the park a lot more, I exercise a lot more and spend more time outdoors which is great	611710-611701-65238969
Regular sleep schedule. Not driving tired.	611710-611701-65340410
Very tired, no energy, been at home for so long, now finding it extremely overwhelming coming back to placement	611710-611701-65339954
Not much choice for indoors activities.	611710-611701-65343532
Noticed my mood swings are more noticeable. Having bouts of mind depression where I sit and do nothing all day when is there is plenty of jobs to do.	611710-611701-65346751
Generally happened around summer so more free time therefore more active lifestyle	611710-611701-65363079
Sleeping patterns worsened unable to get to sleep and stay asleep	611710-611701-65363373
Good life style	611710-611701-65363646
stuck in my room, no where to go but to work	611710-611701-65376195
Being at home more has enabled me to become more proactive	611710-611701-65382930
No	611710-611701-65423303
consuming more alcohol than before and eating fast foods.	611710-611701-65430088
i eat more vegetables now than before and more exercises	611710-611701-65443108
N/a	611710-611701-65471517
During exams, my activity decreased. But since then, I have resumed my normal activity. 3 workouts a week.	611710-611701-65512965
quarantine gave me more time to focus on my health and fitness which im up-keeping now at uni	611710-611701-65559487
Levels of inactivity/activity fluctuated quite dramatically throughout the pandemic, this is true of diet too	611710-611701-65632523
Having more time to myself to focus on fitness	611710-611701-65656449
I have taken more time for myself and started new activities such as yoga to stay mentally healthy during this stressful period	611710-611701-65657915
NO	611710-611701-65684000

No	611710-611701-65754244
having lectures online decreases the amount of activity i do	611710-611701-65953035
I think these questions don't really fully capture the experience over the 6 months. From March to August, I didn't exercise or eat well, I put on significant weight and started to struggle with anxiety and depression. From August, I have been exercising 4 times a week and eating healthily. This is following a course of CBT and as gyms have re-opened.	611710-611701-66240947
Adopted more unhealthy behaviours in terms of sleep cycle.	611710-611701-66753644
I am a lot happier to spend my time at home with my family rather than trying to visit people and go out to entertain the children, I feel less pressured to socialise	611710-611701-66948498
Sleeping patterns have altered, and I am sleeping later. Eating healthier but that is because I wasn't wasting healthily last year anyway.	611710-611701-67046848
I have been comfort eating a lot more which has impacted my mental health, however is a vicious circle as at the time it seems to make me feel happier	611710-611701-67064075
I am very active. I usually go to the gym 6 times a week. During lockdown when gyms were closed it was a struggle.	611710-611701-67189021
I stress eat and have a low metabolism. With limited access to gyms, I have been unable to keep up my fitness.	611710-611701-67254500
no	611710-611701-67272372

13 How has lockdown affected your emotions, feelings and certain aspects of your life?

13.1 13. Sadness

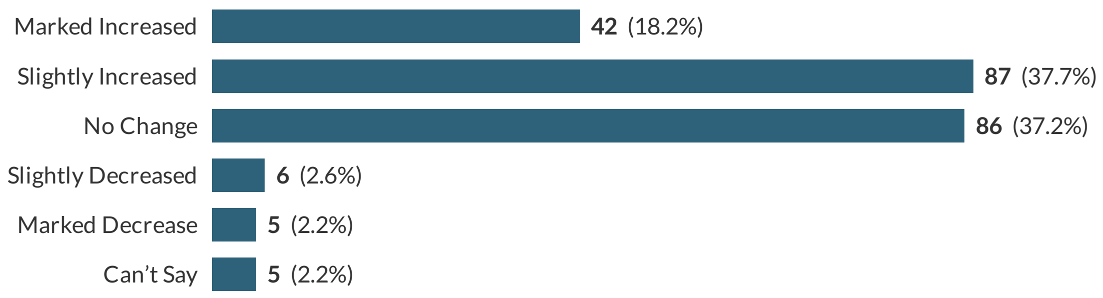

Multi answer: Percentage of respondents who selected each answer option (e.g. 100% would represent that all this question's respondents chose that option)

13.2 14. Irritability

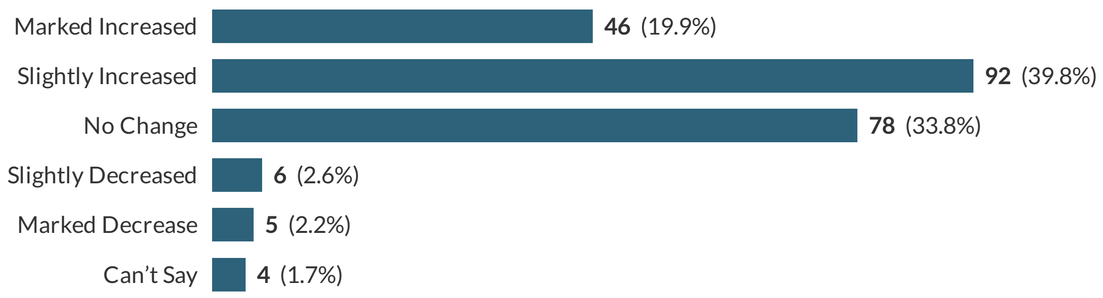

Multi answer: Percentage of respondents who selected each answer option (e.g. 100% would represent that all this question's respondents chose that option)

13.3 15. Fatigue

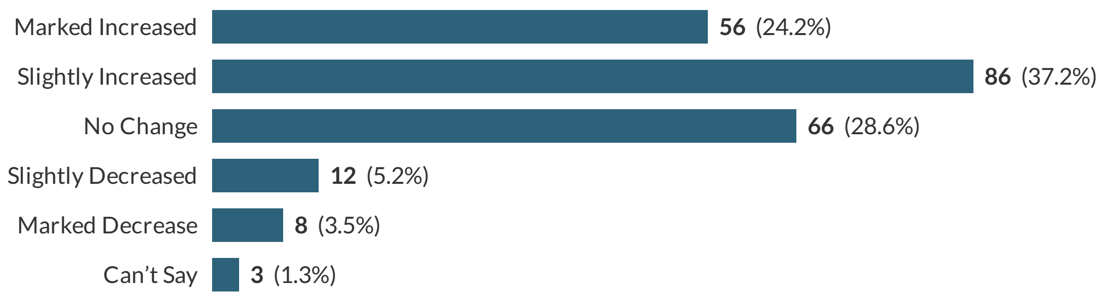

Multi answer: Percentage of respondents who selected each answer option (e.g. 100% would represent that all this question's respondents chose that option)

13.4 16. Frustration

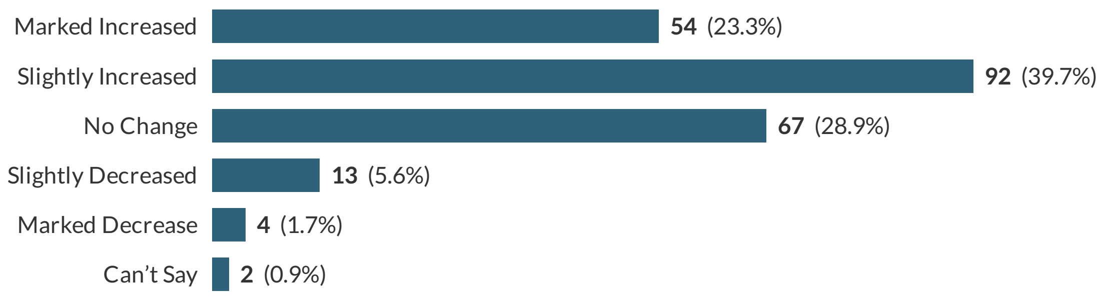

Multi answer: Percentage of respondents who selected each answer option (e.g. 100% would represent that all this question's respondents chose that option)

13.5 17. Loneliness

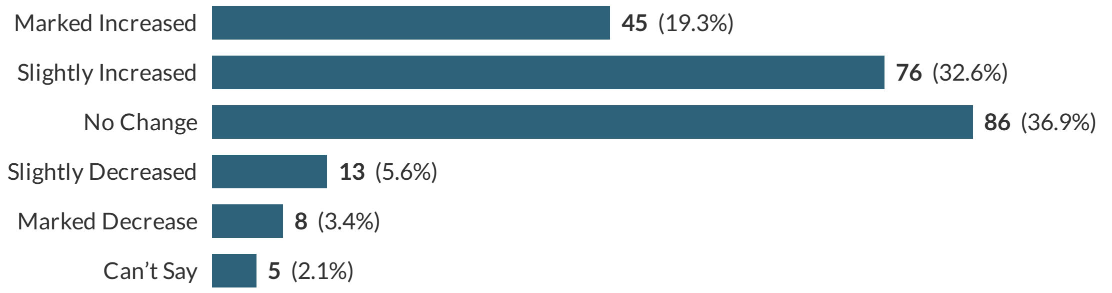

Multi answer: Percentage of respondents who selected each answer option (e.g. 100% would represent that all this question's respondents chose that option)

13.6 18. Sleep

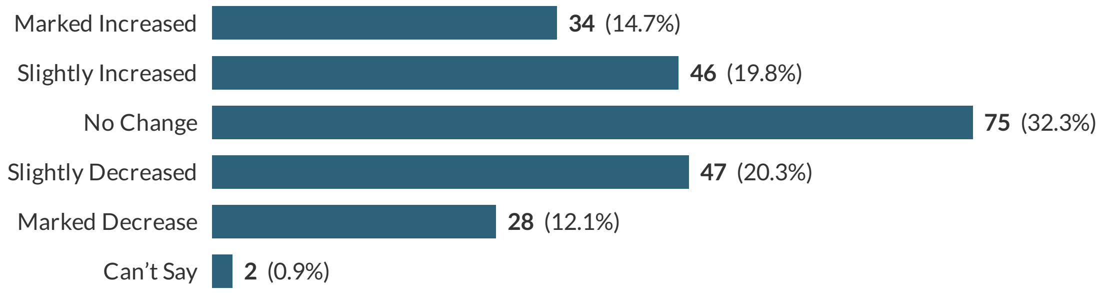

Multi answer: Percentage of respondents who selected each answer option (e.g. 100% would represent that all this question's respondents chose that option)

13.7 19. Appetite

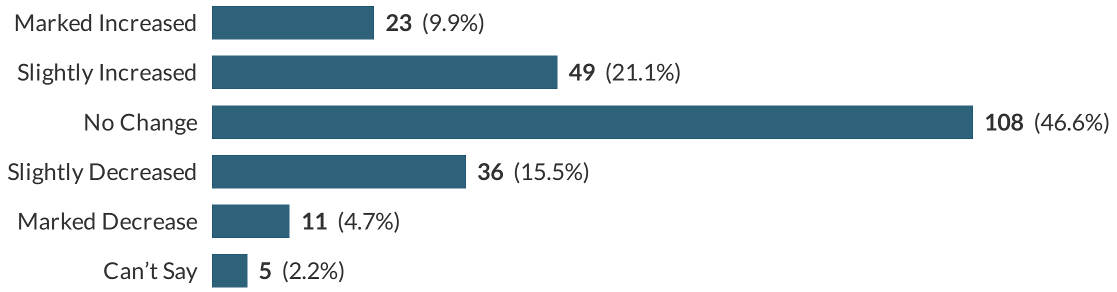

Multi answer: Percentage of respondents who selected each answer option (e.g. 100% would represent that all this question's respondents chose that option)

14 The following questions ask you about your feelings throughout the lockdown. In each case, please answer how often you felt or thought a certain way.

14.1 20. How often have you felt nervous and 'stressed'?

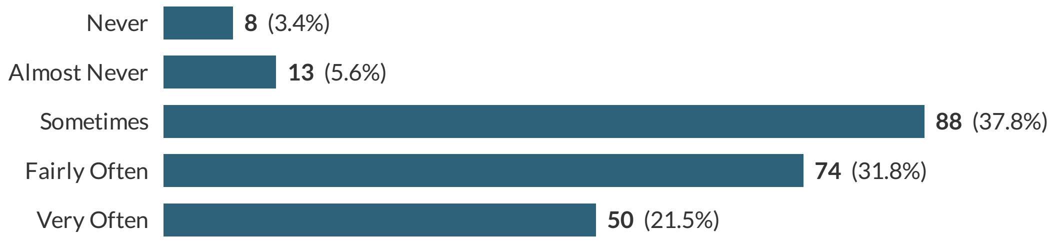

Multi answer: Percentage of respondents who selected each answer option (e.g. 100% would represent that all this question's respondents chose that option)

14.2 21. How often have you felt confident in your ability to handle your personal problems?

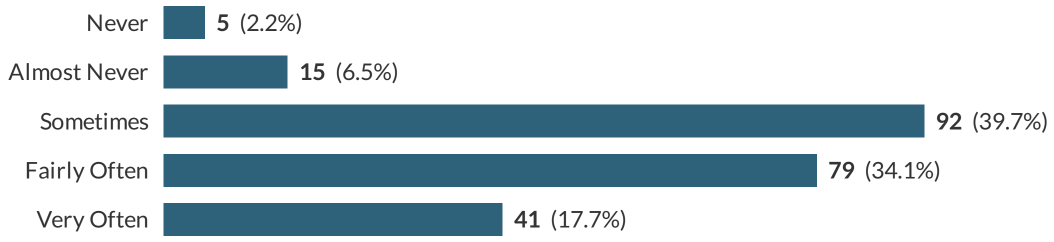

Multi answer: Percentage of respondents who selected each answer option (e.g. 100% would represent that all this question's respondents chose that option)

14.3 22. How often have you found that you could not cope with all the things you had to do?

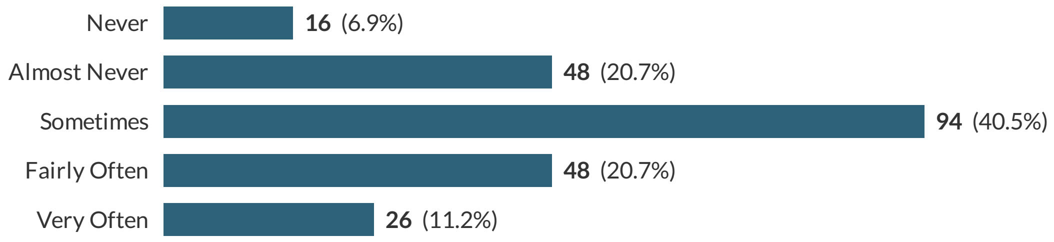

Multi answer: Percentage of respondents who selected each answer option (e.g. 100% would represent that all this question's respondents chose that option)

15 Please respond to the following in terms of how you feel:

15.1 23. Feeling detached from others.

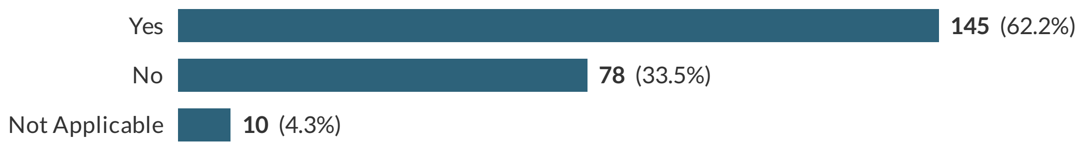

Multi answer: Percentage of respondents who selected each answer option (e.g. 100% would represent that all this question's respondents chose that option)

15.2 24. Poor concentration and felt indecisive.

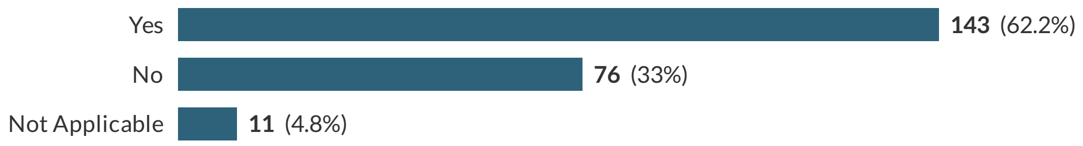

Multi answer: Percentage of respondents who selected each answer option (e.g. 100% would represent that all this question's respondents chose that option)

15.3 25. Deteriorating work performance.

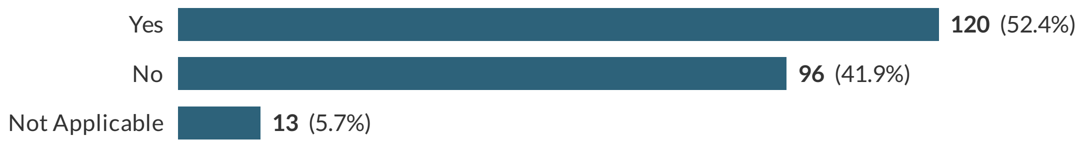

Multi answer: Percentage of respondents who selected each answer option (e.g. 100% would represent that all this question's respondents chose that option)

16 Throughout the lockdown, how often have you been affected by the following problems?

16.1 26. Feeling nervous, anxious, or on edge.

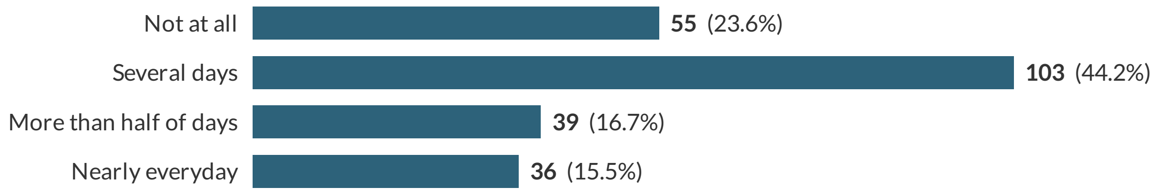

Multi answer: Percentage of respondents who selected each answer option (e.g. 100% would represent that all this question's respondents chose that option)

16.2 27. Worrying too much about different things.

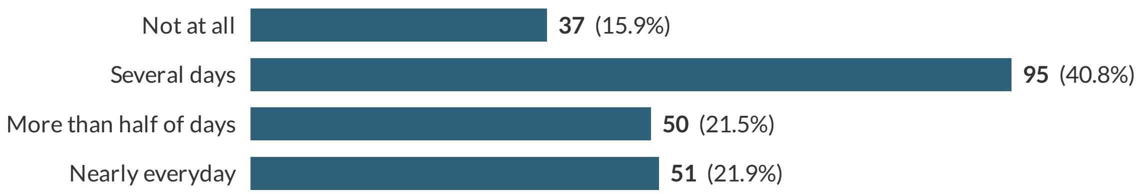

Multi answer: Percentage of respondents who selected each answer option (e.g. 100% would represent that all this question's respondents chose that option)

16.3 28. Trouble relaxing.

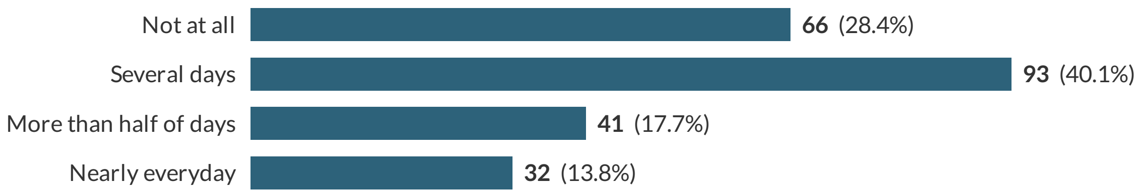

Multi answer: Percentage of respondents who selected each answer option (e.g. 100% would represent that all this question's respondents chose that option)

16.4 29. Becoming easily annoyed or irritable.

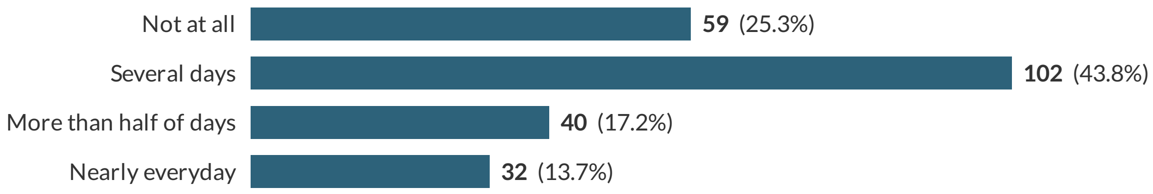

Multi answer: Percentage of respondents who selected each answer option (e.g. 100% would represent that all this question's respondents chose that option)

16.5 30. Feeling afraid, as if something awful might happen.

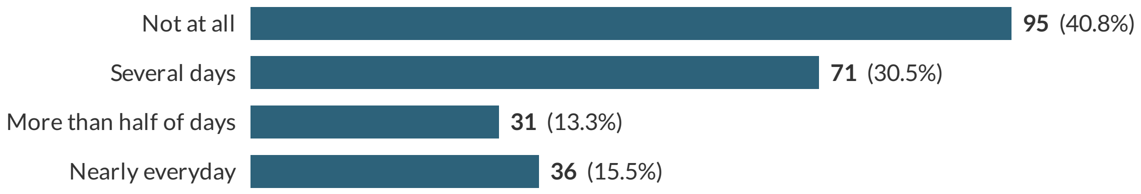

Multi answer: Percentage of respondents who selected each answer option (e.g. 100% would represent that all this question's respondents chose that option)

16.6 31. Not being able to stop or control worrying.

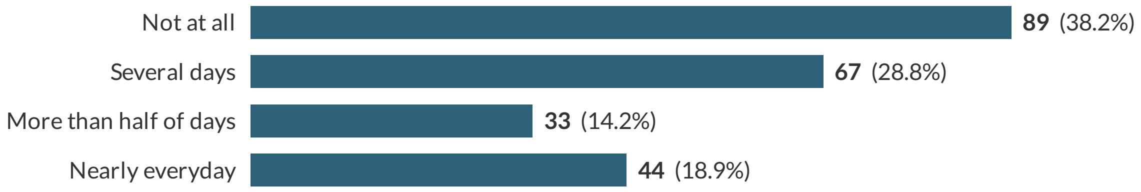

Multi answer: Percentage of respondents who selected each answer option (e.g. 100% would represent that all this question's respondents chose that option)

17 Any other comments regarding mental health:

**Showing all 24 responses**	
Having had COVID-19, I experienced these issues on top of the symptoms which came with the disease.	611710-611701-64913721
I’m usually a strong minded person and so dealt with the pandemic head on by working in the nhs to help so have found it a learning experience for future practice rather than being worried about the virus	611710-611701-64921771
George Floyd and the racism in British society has severely worsened my mental health far worse than Covid	611710-611701-65238969
Normally my family go to work/ school so I am left alone. Having them all home was amazing.	611710-611701-65339765
I am diagnosed with anxiety and previous depression in 2019 and have help from my Dr with medication and counselling sessions to control this	611710-611701-65339824
I do have already existing mental health issues, but they've been more pronounced around lockdown and online learning	611710-611701-65339804
Lockdown enabled greater self care so mental healthy improved	611710-611701-65340410
I have a history of depression and anxiety but had been coping well prior to lockdown. However, due to the pressures of lockdown, I had to be prescribed anxiety medication once again, which I continue to take.	611710-611701-65340848
Treatment from university course in regards to exams with zero responsibility with their reckless marking. Blaming students for poor teaching mental health is dramatically worse since receiving the latest results no accountability from the lecturers no support you need to get it yourself.	611710-611701-65351657
I do have anxiety with ocd tendencies, so the feelings are quite normal for me	611710-611701-65380858

No	611710-611701-65423303
N/a	611710-611701-65471517
my worries were due to other things, not lockdown/covid itself. but being in lockdown gave me more time to think about these other issues which inevitably exacerbated these worries	611710-611701-65559487
Lockdown gave good time to reflect	611710-611701-65656449
the pressure has been too much,I am struggling to complete my degree, i didn't find online tutorials sufficient to receive appropriate support with my dissertation, the lirary was closed and i was resorted to writing my final piece in the crowded café at my local gym, i lost the childcare, i could not gain access to some vital reading material! overwheled!	611710-611701-65677803
NO	611710-611701-65684000
I would have what I would describe as a global anxiety re the Covid situation. I felt guilty about sitting down and working on my thesis as I felt this time was only available through the suffering on individuals and society as a whole.	611710-611701-65695895
No	611710-611701-65754244
I just feel communication on line is not real and sound artificial to me, am not connecting well and afraid of not reaching my grades. An example is when I mistakenly posted my assisnment on time but in the wrong box on turnitin, I have been made known it will be recorded as non submission. That alone worries me so much, its not right.	611710-611701-65797593
I would say it is the first time that I have felt significantly anxious. My sleep was heavily affected at the beginning of the pandemic. I think it was due to their being so many unknowns eg how bad will this be, how will my children be affected, when will i be able to see my family. My studies I would say were more towards the bottom of my 'worry list' which I think is a reflection of the good support we got from ARU. I felt the university did the best they could in such unprecedented times. I remember being in my final lecture before lock down when we got the news that our faculty was closing and this was the first time that I thought things are getting bad.	611710-611701-66034596
As previously mentioned, lock-down and the stress of my PhD caused me to develop moderate anxiety & depression. I have since sought support from the well-being service.	611710-611701-66240947
I have struggled with anxiety and some bouts of depression, i feel lockdown has improved my mental wellbeing a lot, I no longer have regular panic attacks and feel much more able to control my anxiety than before	611710-611701-66948498
I found it has deteriorated generally, but more so after we left lockdown and I realised the effect of lockdown on me. During lockdown I didn’t really feel the effects as I was cushioned by being surrounded by family at home	611710-611701-67046848
no	611710-611701-67272372

18 Please choose one answer for each of the following questions.

18.1 32. Have any of your close contacts tested positive for COVID-19?

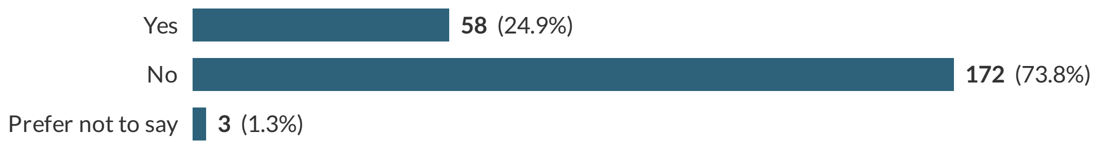

Multi answer: Percentage of respondents who selected each answer option (e.g. 100% would represent that all this question's respondents chose that option)

19 Any other comments:

Showing all 21 responses	
Testing was not widespread at the time of my infection with SARS-CoV-2. NHS 111 told me to isolate (at university) and so I did not get tested; however, the symptoms I had were exactly as described (and more) by the NHS and PHE. My family was safe at home and did not have symptoms.	611710-611701-64913721
Me and my mum	611710-611701-64928259
Lost my mother to covid	611710-611701-65339690
Lost family members and a few close friends to Covid. My friend circle is mostly doctors, and I’ve lost many of them.	611710-611701-65339978
No but I have close family in the shielding category which is a big concern for me, especially with returning to clinical placement.	611710-611701-65340848
90 year old grandad in a care home contracted the virus	611710-611701-65342802
By far the worst university experience, lecturers Always make reference to the fact we are the lesser course as the bsc course is better, the exams were a shambles the question paper needs to be binned questions asked and answered to only find in marking that there were hidden questions in the question. Essays no guidance on writing them, you access library for referencing and then get marked wrong, on checking library you are right. I could not recommend this university for study and will actively discourage people considering study here. Disability discrimination by the university takes place, and because my work place paid for the study you are treated less favourably including being tested for dyslexia.	611710-611701-65351657
My crew mate caught COVID whilst we were at work.	611710-611701-65356103
My friends have and my friends father passed away from COVID	611710-611701-65360204
I had covid syptoms but didn't get tested.	611710-611701-65363373
GR56ONP	611710-611701-65365724
No	611710-611701-65423303
colleague	611710-611701-65430088
N/a	611710-611701-65471517

[illegible]	[illegible]
I was COVID+	611710-611701-65632523
IS COVID 19 REAL AS THE MEDIA PORTRAIT IT TO BE?	611710-611701-65684000
No	611710-611701-65754244
None of my close contacts have taken a test.	611710-611701-66753644
My aunt was hospitalised twice, once initially and the second time as her treatment was unable to shift the fluid from her lungs.	611710-611701-67046848
Some questions were negatively orientated. Perhaps to remove bias. Phrase them in a neutral manner?<br><br>Consider including positives of COVID too?	611710-611701-67046840
no	611710-611701-67272372
